# Early forming label-retaining muscle stem cells require p27^kip1^ for maintenance of the primitive state

**DOI:** 10.1242/dev.100842

**Published:** 2014-04

**Authors:** Joe V. Chakkalakal, Josef Christensen, Wanyi Xiang, Mathew T. Tierney, Francesca S. Boscolo, Alessandra Sacco, Andrew S. Brack

**Affiliations:** 1 Massachusetts General Hospital, Center of Regenerative Medicine, Harvard University, Boston, MA 02114, USA; 2 Faculty of Health and Medical Sciences, University of Copenhagen, 1870 Copenhagen, Denmark; 3 Sanford Children's Health Research Center, Sanford-Burnham Medical Research Institute, La Jolla, CA 92037, USA; 4 Harvard Stem Cell Institute, Boston, MA 02114, USA

**Keywords:** Muscle, Quiescence, Regeneration, Satellite cell, Self-renewal, Stem cell, Mouse

## Abstract

Across different niches, subsets of highly functional stem cells are maintained in a relatively dormant rather than proliferative state. Our understanding of proliferative dynamics in tissue-specific stem cells during conditions of increased tissue turnover remains limited. Using a TetO-H2B-GFP reporter of proliferative history, we identify skeletal muscle stem cell, or satellite cells, that retain (LRC) or lose (nonLRC) the H2B-GFP label. We show in mice that LRCs and nonLRCs are formed at birth and persist during postnatal growth and adult muscle repair. Functionally, LRCs and nonLRCs are born equivalent and transition during postnatal maturation into distinct and hierarchically organized subsets. Adult LRCs give rise to LRCs and nonLRCs; the former are able to self-renew, whereas the latter are restricted to differentiation. Expression analysis revealed the CIP/KIP family members *p21^cip1^* (*Cdkn1a*) and *p27^kip1^* (*Cdkn1b*) to be expressed at higher levels in LRCs. In accordance with a crucial role in LRC fate, loss of *p27^kip1^* promoted proliferation and differentiation of LRCs *in vitro* and impaired satellite cell self-renewal after muscle injury. By contrast, loss of *p21^cip1^* only affected nonLRCs, in which myogenic commitment was inhibited. Our results provide evidence that restriction of self-renewal potential to LRCs is established early in life and is maintained during increased tissue turnover through the cell cycle inhibitor *p27^kip1^*. They also reveal the differential role of CIP/KIP family members at discrete steps within the stem cell hierarchy.

## INTRODUCTION

Efficient stem cell self-renewal and differentiation are essential for adult tissue maintenance and regeneration. Skeletal muscle stem cells, or satellite cells (SCs), are essential for muscle growth and repair (Hutcheson et al., 2009; Lepper et al., 2011; Murphy et al., 2011; Sambasivan et al., 2011). The adult SC pool is a quiescent population of Pax7^+^ cells located at the interface between the basal lamina and muscle fiber that is derived largely from proliferating embryonic/fetal *Pax7^+^/Myod^+^/Myf5^+^/Mrf4* (*Myf6*)*^+^* precursors (Kanisicak et al., 2009; Lepper and Fan, 2010; Biressi et al., 2013). During embryonic development, proliferating Pax7^+^ cells are located in the myotome (at ∼E10.5) and first appear in the SC position during fetal myogenesis (at ∼E16.5) (Relaix et al., 2004, 2005; Kassar-Duchossoy et al., 2005; Sambasivan et al., 2013). During postnatal myogenesis, small subsets of presumptive SC precursors divide less frequently than others (Schultz, 1996). Once muscle growth is completed, the SC pool enters a quiescent state (White et al., 2010). In response to injury, adult quiescent SCs proliferate to produce differentiated progeny for muscle repair and self-renew to repopulate the quiescent SC pool (Shea et al., 2010).

Using cell labeling techniques to monitor cell division history, it has been observed that hierarchically upstream stem cells with long-term self-renewal potential divide less frequently (i.e. retain label) than their downstream progeny (i.e. which dilute label) (Blanpain et al., 2004; Wilson et al., 2008; Foudi et al., 2009). Similarly, SCs with a limited proliferative output are enriched for self-renewal potential (Chakkalakal et al., 2012; Ono et al., 2012; Rocheteau et al., 2012). We recently demonstrated that aged SCs that retained H2B-GFP label [label-retaining cells (LRCs)] possess extensive self-renewal potential in aged muscle, whereas cells that undergo more divisions and lose label [non-label-retaining cells (nonLRCs)] precociously differentiate and are functionally limited (Chakkalakal et al., 2012). Moreover, aged LRCs were enriched for *Spry1*, an intracellular inhibitor of the potent mitogen Fgf2 and a crucial regulator of SC reversible quiescence (Shea et al., 2010; Chakkalakal et al., 2012). Together, these findings indicate that LRCs possess molecular brakes that restrict entry into the cell cycle. Cell cycle inhibitors are well known modulators of stem cell fate. For example, in hematopoietic stem cells (HSCs), loss of cell cycle inhibitors of the CIP/KIP family, such as p57^kip2^ (Cdkn1c) and p27^kip1^ (Cdkn1b), affects stem cell function and self-renewal capability (Cheng et al., 2000; Matsumoto et al., 2011; Zou et al., 2011). Studies of the CIP/KIP family in muscle progenitors demonstrate their importance in terminal differentiation (Halevy et al., 1995; Parker et al., 1995; Zhang et al., 1999b; Hawke et al., 2003; Messina et al., 2005). However, their role in muscle stem cell quiescence and cell fate decisions has not been examined.

Despite the importance of label-retaining stem cells, it is not known how and when functionally distinct LRCs and nonLRCs within the SC pool are formed, and whether these distinct populations can be revealed in contexts of increased skeletal muscle turnover such as growth, regeneration and disease. In the present study, we find that subsets of LRCs and nonLRCs can be identified shortly after birth. However, functional distinctions between LRCs and nonLRCs only become readily apparent at later stages of postnatal maturation. Loss-of-function studies reveal an essential role for p27^kip1^ in the maintenance of the primitive state and self-renewal potential of LRCs, and thereby adult SC pool size during tissue turnover.

## RESULTS

### Formation of label-retaining and non-label-retaining SCs

**Fig. 1.**
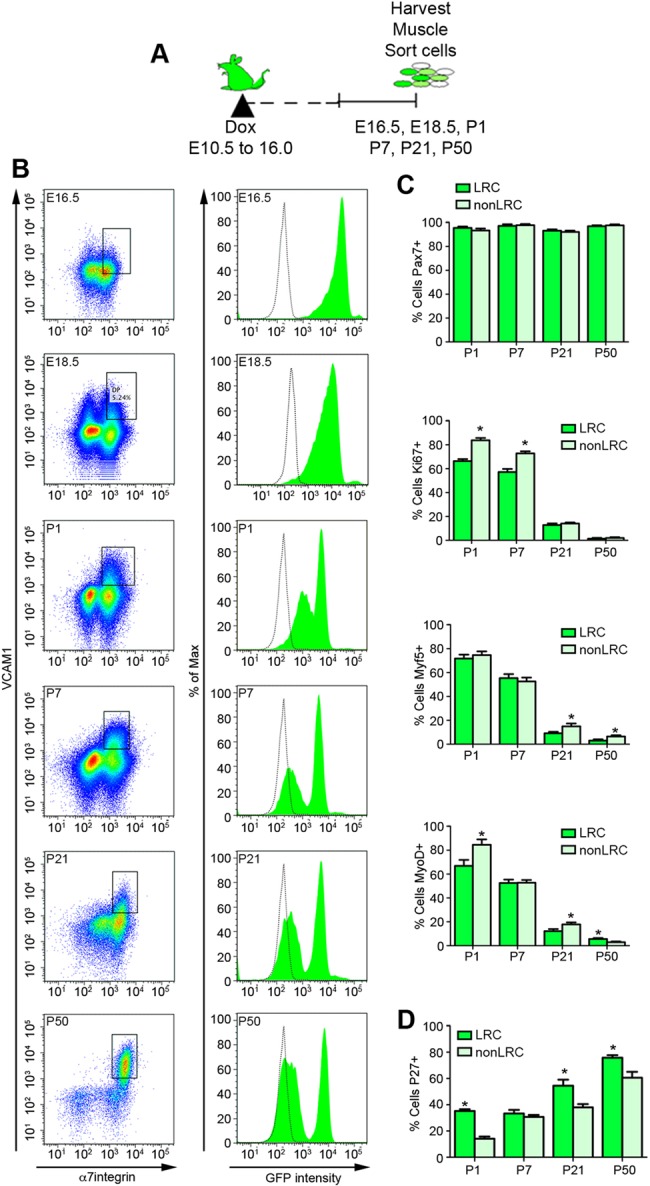
**H2B-GFP labeling reveals the formation of LRC and nonLRCs at birth.** (A) Timecourse of Dox feeding (E10.5-16) and chase and SC harvest periods of TetO-H2B-GFP mice. (B) SC sort profile and distribution of H2B-GFP intensity at different chase time points (E16.5 represents no chase). Vehicle-treated TetO-H2B-GFP control is in gray. (C) Percentage of sorted LRCs and nonLRCs expressing Pax7, Ki67, MyoD or Myf5 at different neonatal and postnatal stages. (D) Percentage of sorted p27^kip1^^+^ LRCs and nonLRCs at different neonatal and postnatal stages. (C,D) Data were averaged (*n*=3-4 mice per group) and expressed as mean±s.e.m. (*t*-tests, *P<0.05, LRC versus nonLRC).

We previously used doxycycline (Dox)-inducible TetO-H2B-GFP reporter mice to quantify the proliferative history of SCs during aging (Chakkalakal et al., 2012). To date, it remains unknown whether H2B-LRCs and H2B-nonLRCs (hereafter referred to as LRCs and nonLRCs) are born early in life or emerge as a feature of aging. To this end, we pulsed TetO-H2B-GFP mouse embryos with Dox from E10.5-16 to induce transient H2B-GFP expression and chased by Dox withdrawal throughout postnatal maturation (Fig. 1A). Upon Dox withdrawal, the H2B-GFP label is diluted in proportion to cell division (Foudi et al., 2009). Myogenic cells were analyzed for H2B-GFP expression by FACS analysis of Vcam1^+^, integrin α7^+^, Sca1 (Ly6a)^−^, CD31 (Pecam1)^−^/CD45 (Ptprc)^−^, propidium iodide (PI)^−^ cell populations (Fig. 1B; supplementary material Fig. S1A) (Brohl et al., 2012; Chakkalakal et al., 2012).

At E16.5 (no chase), ∼90% of isolated cells were Pax7^+^, of which 100% were H2B-GFP^+^ (Fig. 1B; supplementary material Fig. S1B and Fig. S2B). Two days later (E18.5), the average H2B-GFP intensity of the SC pool was lower and centered around a broad single distribution. At P1, the average H2B-GFP intensity of the SC pool had decreased further and the distribution had become heterogeneous. Based on a 50% (0.33 log_10_) decline in H2B-GFP levels with every cell division (supplementary material Fig. S2F,G), ∼40% of the SC pool undergoes ∼2 divisions (LRCs), whereas the remainder undergo ∼4 divisions (nonLRCs) between E16.5 and P1. Therefore, LRCs and nonLRCs are established at birth. During the first week of postnatal life, H2B-GFP intensity levels decline in nonLRCs while remaining constant in LRCs. Throughout the remainder of postnatal maturation, the mean H2B-GFP intensity within the LRC and nonLRC subsets does not shift; however, the proportion of nonLRCs increases and that of LRCs decreases. Between P7 and P50, the fraction of LRCs decreases from 52% to 36% of the SC pool. To confirm that levels of H2B-GFP in SCs were not influenced by variable expression of the surface markers Vcam1 and integrin α7 during ontogeny (Brohl et al., 2012), we included an unbiased approach to obtain Pax7^+^ SCs with subsequent H2B-GFP intensity quantification using magnetic-activated cell sorting, then stained with anti-Pax7 and analyzed for H2B-GFP levels (supplementary material Fig. S1C). Together, these results demonstrate that the adult SC pool is composed of LRCs and nonLRCs that can be observed at birth.

SCs undergo a coordinated program during proliferation and lineage progression, initially expressing Myf5 and MyoD during proliferation, followed by a decrease in Pax7 and increase in myogenin (MyoG) as they differentiate (Olguin and Olwin, 2004; Zammit et al., 2004; Olguin et al., 2007). We tested the proliferative state of LRCs and nonLRCs isolated throughout ontogeny (Fig. 1C; supplementary material Fig. S1E). Pax7 expression did not differ between LRCs and nonLRCs throughout ontogeny; however, during the first week of postnatal life nonLRCs expressed a larger fraction of cycling markers (MyoD, Ki67) and a smaller fraction of quiescent markers (such as p27^kip1^) compared with LRCs (Fig. 1C,D; supplementary material Fig. S1E-G). Therefore, LRCs were in a more dormant state. At ∼3 weeks after birth, analysis of the proliferative state using short-term EdU pulse labeling and immunostaining for MyoD/Myf5/MyoG reveals that nonLRCs have delayed entry into quiescence (Fig. 1C; supplementary material Fig. S1D,E). In adult muscle, nearly all Pax7^+^ LRC and nonLRC subsets were MyoD^−^/Myf5^−^/Ki67^−^, suggesting that they had returned to the homeostatic quiescent state commonly observed in adult muscle. Together, these findings suggest that, during early postnatal muscle growth, LRCs are set aside into a quiescent state, followed by the eventual transition of nonLRCs into quiescence as muscle growth ceases.

### Re-establishing LRCs and nonLRCs in response to injury

**Fig. 2.**
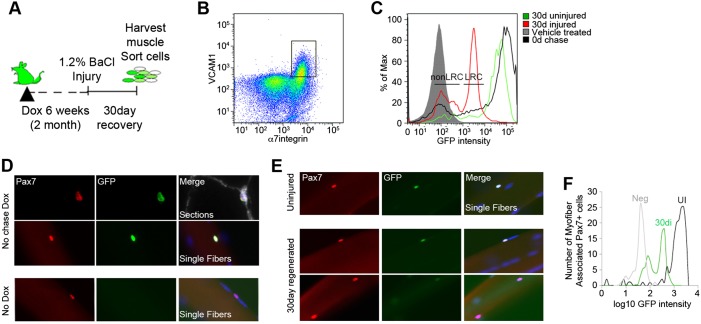
**H2B-GFP labeling reveals the re-establishment of LRCs and nonLRCs in response to injury.** (A) Dox feeding and injury paradigm with adult TetO-H2B-GFP mice. (B) Representative SC sort profile of 6-week pulsed or 30-day post-injury muscle. (C) Representative distribution of H2B-GFP intensity from sorted SCs harvested 30 days post-injury (red) or from uninjured contralateral muscle (green). No-chase H2B-GFP profile isolated from Dox-fed TetO-H2B-GFP mice (black). H2B-GFP intensity profile from vehicle-fed TetO-H2B-GFP mice (gray filled line). Two discrete populations (LRC and nonLRC) of SCs form after injury. To determine the fraction of LRCs and nonLRCs within FACS isolated SCs, we created positive selection gates at the boundaries where the cell numbers reach a minimum across the total H2B-GFP intensity. The fraction of the total population within each gate was categorized as LRC or nonLRC (see Materials and Methods for more detail). (D) Transverse sections (top) and single fibers (middle) from Dox-fed no-chase TetO-H2B-GFP mice show GFP expression in Pax7^+^ SCs. H2B-GFP was not detected in Pax7^+^ cells from vehicle-treated TetO-H2B-GFP mice (bottom row). (E) H2B-GFP label retention in Pax7^+^ cells from single fibers in uninjured and regenerated muscle (30 days after injury). (F) Profile of H2B-GFP expression in uninjured (black) or 30-day regenerated (green) single muscle fiber-associated SCs; vehicle-treated H2B-GFP provided a negative control (gray). H2B-GFP profiles were collected from 6-8 mice per group.

We next asked whether LRC and nonLRC subsets are re-established in niche-repopulating Pax7^+^ SCs after injury. Rather than using embryonically pulsed TetO-H2B-GFP adult mice, which would lose all label in the nonLRC population upon injury, adult TetO-H2B-GFP mice were fed Dox, injured and left to recover for 30 days (Fig. 2A). After 6 weeks of Dox feeding (0-day chase), a high proportion (95%) of SCs and non-myogenic cells robustly expressed H2B-GFP (Fig. 2B-D; supplementary material Fig. S2A-C). In uninjured adult muscle, after 30 days of chase, the SC pool had diluted H2B-GFP label corresponding to 0-2 divisions (Fig. 2C) (Chakkalakal et al., 2012). H2B-GFP could not be detected in myonuclei of uninjured muscle (Fig. 2D; supplementary material Fig. S2E)*.* In regenerated muscle, H2B-GFP^+^ SCs contribute to the myonuclei of regenerated muscle fibers (supplementary material Fig. S2D,E). Analysis of the SC pool revealed that the distribution of H2B-GFP was heterogeneous; a subset that constitutes ∼56% of the repopulating SC pool undergoes 3-5 divisions (LRCs), whereas the remaining SCs undergo 6 or more divisions (nonLRCs) (Fig. 2C). In support, two distinct H2B-GFP intensity populations were observed in Pax7^+^ SCs from central nucleated single muscle fibers from regenerated muscles (Fig. 2E,F). However, both populations were Pax7^+^/MyoD^−^, confirming that all niche-repopulating SCs return to quiescence after injury (supplementary material Fig. S2C) (Shea et al., 2010).

To confirm that levels of H2B-GFP reflect proliferative output, we cultured SCs from Dox-treated TetO-H2B-GFP mice in either high (20%) or low (3%) serum conditions to induce cell division or cell cycle exit, respectively. In high serum conditions the majority of SC progeny lose label after 8 days in culture (supplementary material Fig. S2F). By contrast, activated SCs incubated in low serum conditions for 4 days maintained high levels of H2B-GFP expression in multinucleated myotubes and Pax7^+^ cells (supplementary material Fig. S2H). In addition, we devised a strategy to follow H2B-GFP levels in terminally differentiated nuclei of mature muscle fibers (supplementary material Fig. S2I). The results show that H2B-GFP dilution in terminally differentiated muscle fiber nuclei is significantly lower than estimates of turnover of SCs in adult uninjured muscle. Together, these observations demonstrate that changes in H2B-GFP expression can be used as a readout of proliferative history of SCs and their progeny.

### LRC subsets maintain a more primitive phenotype during proliferation

**Fig. 3.**
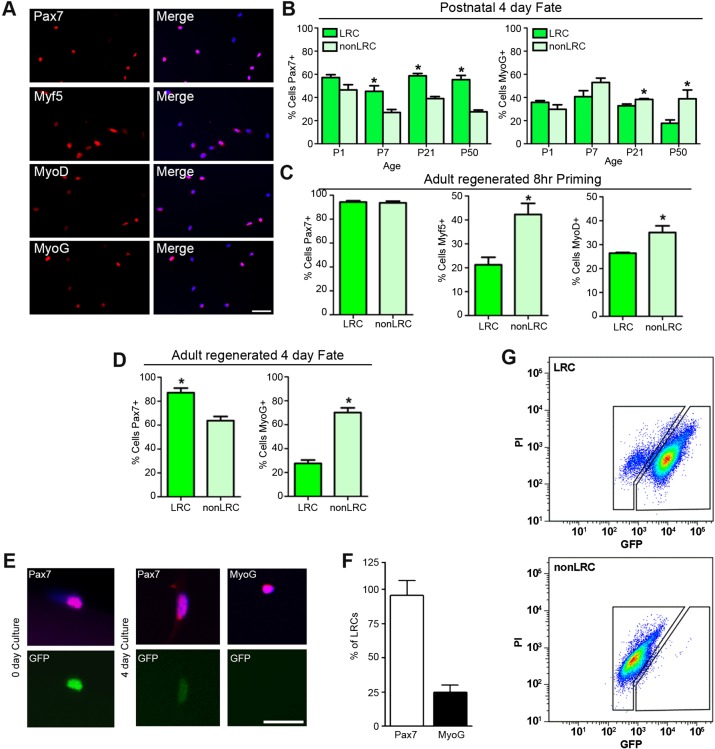
**Proliferative history and cell fate become coupled during postnatal maturation.** (A) Representative Pax7, Myf5, MyoD and MyoG immunostaining alone or in combination with DAPI staining (merge) of sorted LRCs and nonLRCs from TetO-H2B-GFP mice cultured for 4 days in plating medium. Scale bar: 50 µm. (B) Percentage of sorted LRCs or nonLRCs isolated throughout ontogeny expressing Pax7/MyoG after 4 days in culture. (C,D) Percentage of sorted LRCs or nonLRCs isolated from 30-day regenerated adult muscle and plated for 8 h (C, priming) or 4 days (D, fate) expressing Pax7, Myf5, MyoD or MyoG. (B-D) Data were averaged (*n*=3 mice per group) and expressed as mean±s.e.m. (*t*-tests, *P<0.05, LRC versus nonLRC). (E) LRCs stained with anti-Pax7 and anti-MyoG after 0 or 4 days in culture. Scale bar: 20 μm. (F) Percentage of Pax7^+^ or MyoG^+^ cells that retained GFP label after 4 days in culture. Data were averaged (*n*=3 mice per group) and expressed as mean±s.e.m. (*P*<0.05). (G) H2B-GFP intensity of sorted adult LRCs and nonLRCs that had been plated, treated with Dox on day 1 and cultured in plating medium for 9 days. LRC cultures form LRC and nonLRC populations, whereas cultured nonLRCs divide as a single population.

After entry into the cell cycle, SCs are able to self-renew or differentiate. We assessed the lineage bias of LRCs and nonLRCs throughout ontogeny and after injury, based on myogenic lineage markers of self-renewal potential (Pax7), lineage commitment (MyoD/Myf5) and differentiation (MyoG) (Fig. 3A). After 4 days in culture, the fraction of SCs from neonatal muscle (P1) expressing Pax7, MyoD, Myf5 and MyoG was indistinguishable between LRC and nonLRC subsets (Fig. 3B). These results suggest that whereas the proliferative history of neonatal SCs is heterogeneous, the cell fate potential of LRCs and nonLRCs is similar. By contrast, LRCs isolated at later stages of muscle maturation (P21 onwards) maintain a more primitive phenotype during proliferation, as compared with nonLRCs (Fig. 3B). Next, quiescent LRCs and nonLRCs isolated from regenerated muscle (30 days post-injury) were cultured for 8 h or 4 days. After 8 h in culture, an increased fraction of nonLRCs was MyoD^+^/Myf5^+^ compared with LRCs, consistent with a more rapid entry into the myogenic program (Fig. 3C). After 4 days in culture, LRCs retained a high proportion of Pax7^+^ cells (∼90%); by contrast, nonLRCs lost Pax7 expression and turned on MyoG (Fig. 3D). No differences in MyoD and Myf5 expression between LRCs and nonLRCs were observed (supplementary material Fig. S3A). Importantly, *in vitro* analysis demonstrates that H2B-GFP^+^ and rare unlabeled H2B-GFP^−^ SCs isolated after 6 weeks of Dox loading are functionally indistinguishable (supplementary material Fig. S3B). In conclusion, similar to postnatal muscle, LRCs isolated from regenerated adult muscle maintain a more primitive phenotype during proliferation *in vitro*.

To examine the potential for interconversion, LRCs and nonLRCs were collected by FACS, re-fed Dox for 24 h, cultured in plating medium for 9 days and re-analyzed for H2B-GFP levels by FACS (Fig. 3G). The results demonstrate that LRC cultures formed LRC and nonLRC populations. By contrast, the H2B-GFP intensity of nonLRC cultures was distributed as a single population. In addition, analysis of Pax7 and MyoG expression in 4-day cultured LRCs demonstrated that 95% of Pax7^+^ cells retained H2B-GFP, whereas 75% of MyoG^+^ cells diluted H2B-GFP label (Fig. 3E,F), suggesting that LRCs undergoing more divisions, i.e. transitioning to nonLRCs, are biased to differentiate. Moreover, LRCs cultured for 4 days and switched to low serum conditions are fully capable of terminally differentiating into multinucleated myotubes (supplementary material Fig. S3D). Together, these results show that LRCs can maintain themselves and give rise to nonLRCs, the latter being differentiation competent.

### Stem cell potential of LRCs and nonLRCs

**Fig. 4.**
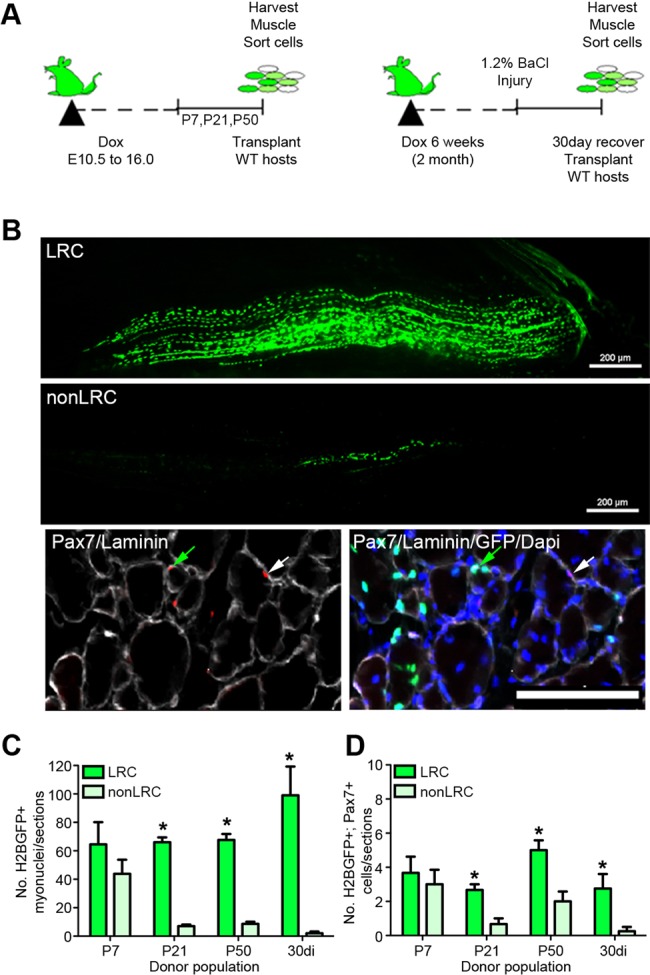
**LRCs and nonLRCs are hierarchically organized and functionally distinct.** (A) Schematic depicts isolation of 2000 LRCs and 2000 nonLRCs for transplant. (B) Representative whole-mount of regenerated (30 days post-transplantation) host TA muscle after engraftment of LRCs (top) or nonLRCs (bottom). Transverse sections of host muscle stained for Pax7 or laminin or with DAPI. Muscles contain donor (GFP^+^, green arrow) and host-derived (GFP^−^, white arrow) Pax7^+^ cells underneath basal lamina and sublaminar myonuclei. Scale bars: 200 μm. (C,D) Number of donor-derived GFP^+^ myonuclei (C) and Pax7^+^/GFP^+^ (D) sublaminar cells. GFP^+^ cell numbers were summed over 200 μm of regenerated muscle. Data were averaged (*n*=3 mice per group) and expressed as mean±s.e.m. (*t*-tests, *P<0.05).

We next examined the transplantation potential of LRCs and nonLRCs. To this end, LRCs and nonLRCs were isolated from P7 to P50 and 30-day regenerated adult muscle and injected into pre-injured wild-type (host) mice, which were then allowed to recover for 30 days (Fig. 4A). During the time of recovery, hosts were fed Dox to maintain H2B-GFP expression in donor-derived SCs. Quantitative analysis revealed that the extent of myonuclear contribution (Fig. 4B,C) and the number of sublaminar Pax7^+^/GFP^+^ cells (Fig. 4B,D) was greater in transplants of LRCs than nonLRCs, beginning at 3 weeks of life and persisting in adult uninjured and regenerated muscle. By contrast, LRCs and nonLRCs from P7 pups were indistinguishable. Indeed, P7 nonLRCs possess engraftment potential that is lost abruptly during postnatal maturation. Taken together, these data support a model whereby, shortly after birth, the SC pool is composed of functionally equivalent LRCs and nonLRCs that transition into a unidirectional hierarchical relationship throughout ontogeny.

### A shift in stem cell proliferative character biases cell fate decisions in muscular dystrophy

**Fig. 5.**
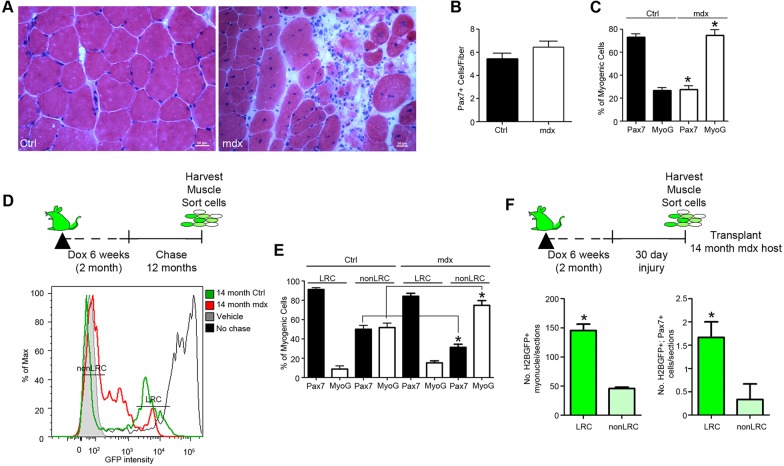
**SC proliferative history during mdx disease progression.** (A) Representative histological stains of TA muscles from 14-month control and mdx mice. Scale bars: 50 μm. (B) Number of Pax7^+^ SCs per single muscle fiber from 14-month control and mdx mice. (C) Percentage of sorted SCs from 14-month control and mdx mice expressing Pax7 or MyoG after 4 days in culture. (D) Dox feeding and chase strategy in H2B control and H2B mdx mice. Representative FACS profiles of H2B-GFP intensity in SCs from 14-month chased control (green) and mdx (red) mice. (E) Percentage of sorted LRCs and nonLRCs expressing Pax7 or MyoG^+^ after 4 days in culture. (F) Schematic and number of donor-derived GFP^+^ myonuclei (left) and Pax7^+^/GFP^+^ (right) sublaminar cells in host 14-month-old mdx muscle. GFP^+^ cell numbers were summed over 200 μm of regenerated muscle. (C,E,F) Data were averaged (*n*=3 mice per group) and expressed as mean±s.e.m. (*t*-tests, *P<0.05).

In mdx mice, a genetic model of Duchenne muscular dystrophy (DMD), SCs initially form and function normally, but eventually lose self-renewal potential and instead ectopically differentiate (Yablonka-Reuveni and Anderson, 2006). We used the mdx model to study the relationship between LRCs and nonLRCs under chronic regeneration. We initially analyzed 14-month mdx mice, an age when there is significant muscle degeneration, no change in SC number but a preference to differentiate (Fig. 5A-C). Adult (2-month) male (mdx) and female (control) TetO-H2B-GFP littermates received Dox for 6 weeks and were either analyzed immediately (0-day chase) or chased for 12 months (Fig. 5D). Surprisingly, in spite of the degenerative context of the mdx mouse, the SC pool contains a small LRC population, which appears to have undergone 1-4 divisions during a 12-month chase and therefore does not differ from control muscle (Fig. 5D). However, the relative fraction of SCs within each population is shifted, with a higher proportion of SCs from mdx mice contained in the nonLRC subset compared with controls. Therefore, during disease progression, nonLRCs predominate at the expense of LRCs.

We next tested the lineage bias of LRCs and nonLRCs in dystrophic muscle. Similar to control SCs, LRCs from TetO-H2B-GFP;mdx muscle maintained Pax7 expression *in vitro*, whereas nonLRCs turned on MyoG (Fig. 5E; supplementary material Fig. S4). By contrast, nonLRC cultures from mdx mice had a higher fraction of differentiated myogenic cells than control nonLRC cultures, suggesting that disease progression promotes the loss of LRCs with high self-renewal potential, in favor of differentiation-prone nonLRCs. However, small subsets of SCs are LRCs and retain self-renewal potential in pathogenic muscle, implicating a cell-autonomous property of LRCs to maintain self-renewal potential. To examine this issue further, we transplanted LRCs and nonLRCs isolated from regenerated control muscle into 14-month-old mdx hosts (Fig. 5F). As anticipated, the number of Pax7^+^ cells and myonuclei derived from LRC H2B-GFP^+^ SCs was higher than for nonLRCs. Interestingly, the self-renewal and differentiation potential of LRCs in mdx hosts was similar to that of control hosts. These results demonstrate that LRCs retain self-renewal potential in a degenerated environment, similar to that observed in aged muscle (Chakkalakal et al., 2012).

### The cell cycle inhibitors p27^kip1^ and p21^cip1^ are enriched in LRCs

**Fig. 6.**
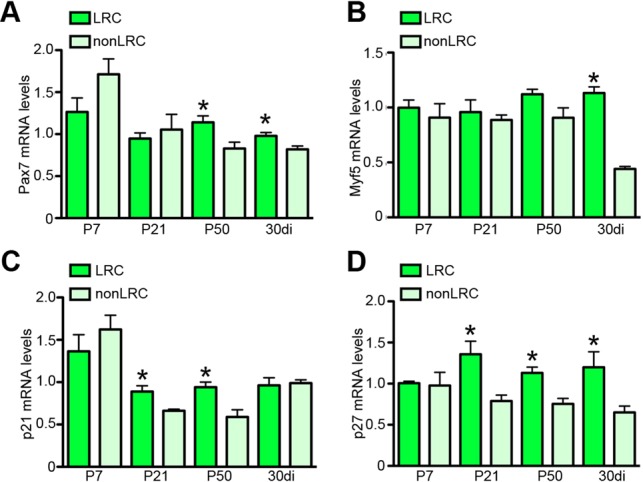
***p27^kip1^* is enriched in LRCs throughout growth and regeneration.** Expression of myogenic fate markers (A, *Pax7*; B, *Myf5*) and cell cycle inhibitors (C, *p21^cip1^*; D, *p27^kip1^*) in LRCs and nonLRCs during ontogeny or after injury. Expression values were normalized to the corresponding gene expression level in LRCs at the P7 time point. The normalization within the P7 time point was obtained by setting the first of four replicates to 1. Data were averaged (*n*=3-4 mice per group) and expressed as mean±s.e.m. (*t*-tests, *P<0.05).

To decipher regulators of LRCs and nonLRCs that control the proliferative output and lineage commitment of SCs, we analyzed transcriptional readouts of the myogenic lineage (*Pax7*, *Myf5* and *Myod*) (Fig. 6A,B; supplementary material Fig. S5A) and cell cycle genes implicated in quiescence [*p27^kip1^*, *p21^cip1^* (*Cdkn1a*), *p57^kip2^*] (Fig. 6C,D; supplementary material Fig. S5B) in LRCs and nonLRCs during ontogeny and after injury. Three weeks after birth, when LRCs and nonLRCs are functionally distinct, we observed an enrichment of the quiescence genes *p27^kip1^* and *p21^cip1^* (Fig. 6C,D; supplementary material Fig. S5B). By contrast, the myogenic genes *Pax7*, *Myf5* and *Myod* were not differentially expressed (Fig. 6A,B; supplementary material Fig. S5A). *p57^kip2^*, a functionally related family member to *p27^kip1^*, was barely detectable in postnatal and adult LRC and nonLRC populations (supplementary material Fig. S5B). As mice reach adulthood, *Pax7* and *Spry1* become enriched in LRCs (Fig. 6A; supplementary material Fig. S5A). After injury, when LRCs and nonLRCs are re-established, LRCs are enriched for *Pax7*, *p27^kip1^* and *Myf5* compared with nonLRCs (Fig. 6A,B,D). Therefore, the onset of functional differences between LRCs and nonLRCs coincides with the upregulation of both *p27^kip1^* and *p21^cip1^* in LRCs.

### p27^kip1^ is required for the maintenance of self-renewing LRCs

**Fig. 7.**
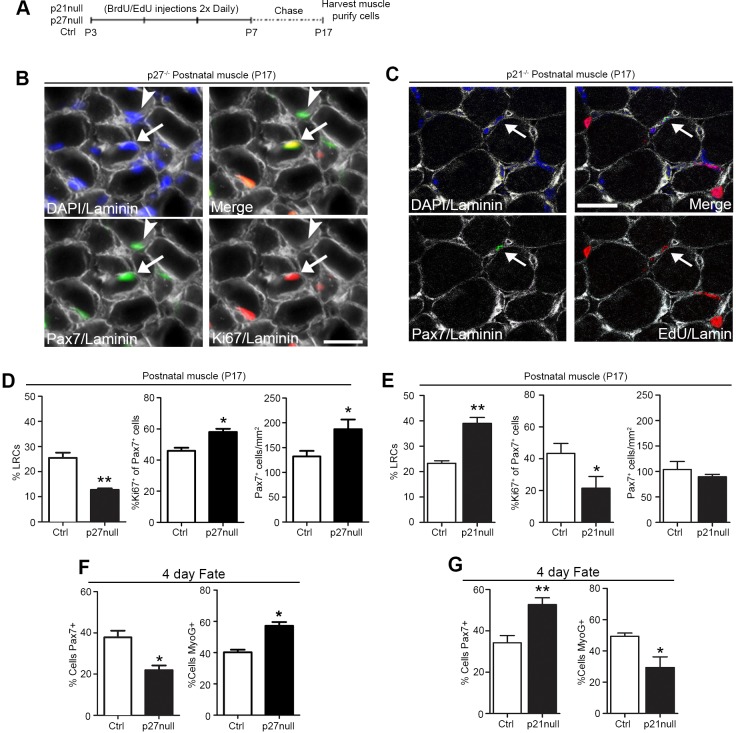
***p27^kip1^* is required for the maintenance of self-renewing LRCs.** (A) Outline of experiments to assess BrdU/EdU label retention. (B) Representative images of Pax7^+^ and Ki67^+^ cells from postnatal *p27* null muscle. Arrowhead and arrow indicate Pax7^+^/Ki67^−^ and Pax7^+^/Ki67^+^ SCs, respectively. Scale bar: 20 μm. (C) Representative images of Pax7^+^ and EdU^+^ cells from postnatal *p21* null muscles. Arrow indicates Pax7^+^/EdU^+^ SCs. Scale bar: 100 μm. (D) Postnatal (P17) *p27* null and Ctrl muscles and (E) postnatal (P17) *p21* null and Ctrl muscle analyzed for label-retaining (BrdU^+^/EdU^+^) SCs (left), the percentage of Ki67^+^ SCs (middle) and the number of SCs per serial muscle section (right). (F) Percentage of Pax7^+^/MyoG^+^ sorted SCs from *p27* null and Ctrl muscles and (G) *p21* null and Ctrl muscles after 4 days in culture. (D-G) Data were averaged (*n*=3-4 mice per group) and expressed as mean±s.e.m. (*t*-tests, *P<0.05, **P<0.01).

Owing to the pattern of consistently higher *p27^kip1^* and *p21^cip1^* expression in LRCs (Fig. 6C,D; supplementary material Fig. S5B), we examined whether either is required to maintain LRCs *in vivo* throughout postnatal maturation. BrdU has previously been used to identify label-retaining stem cells in blood and muscle (Shinin et al., 2006; Wilson et al., 2008; Rocheteau et al., 2012). Control (Ctrl), *p27* null and *p21* null (*p27^kip1^* and *p21^cip1^* germline knockout) neonatal mice received BrdU for 5 days followed by a 10-day chase (Fig. 7A,C; supplementary material Fig. S6A). The fraction of BrdU-labeled LRCs (BrdU-LRCs) in *p27* null muscle was reduced compared with Ctrl (Fig. 7A,D; supplementary material Fig. S6A). In addition, the fraction of proliferating (Ki67^+^) and total number of Pax7^+^ cells was increased in *p27* null muscle compared with Ctrl (Fig. 7A,B,D). Together, these observations suggest that p27^kip1^ is required for the maintenance of BrdU-LRCs during postnatal growth. By contrast, the fraction of BrdU-LRCs was increased in *p21* null muscle compared with Ctrl; moreover, a decline in the percentage of proliferating Pax7^+^ cells was observed in *p21* null muscle (Fig. 7A,C,E; supplementary material Fig. S6B). No change in SC numbers was found (Fig. 7E; supplementary material Fig. S6B). Analysis of postnatal muscle growth shows that muscle fiber size was smaller in juvenile *p27* null and *p21* null relative to Ctrl mice (supplementary material Fig. S6D-F). Overall, these data demonstrate that p27^kip1^ is required for the maintenance of BrdU-LRCs during postnatal growth. By contrast, p21^cip1^ negatively regulates the number of BrdU-LRCs.

**Fig. 8.**
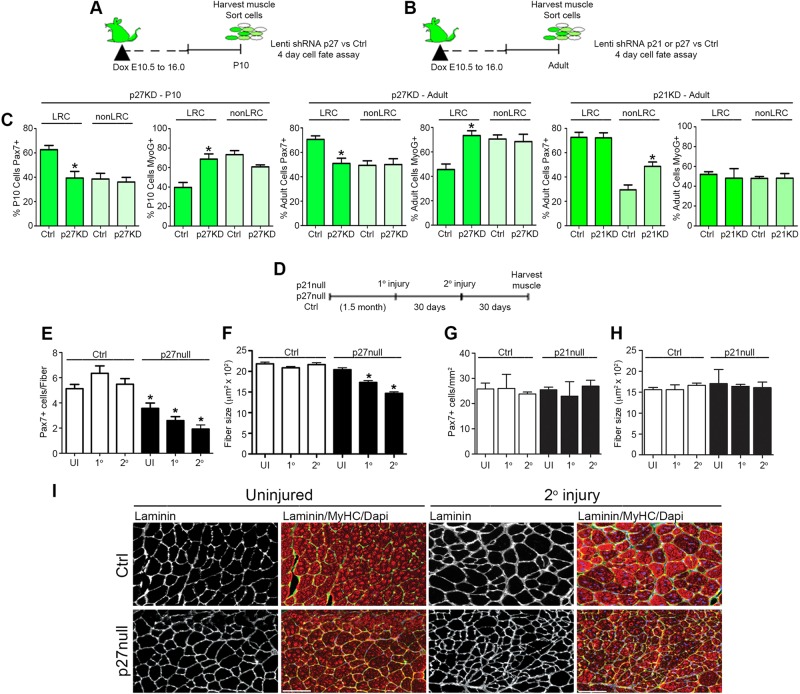
**p27^kip1^ function is restricted to LRCs.** Lentiviral shRNA strategy to knockdown p27^kip1^ (p27KD) in postnatal (P10) LRCs or nonLRCs (A) or to knockdown p21^cip1^ (p21KD) or p27^kip1^ in adult LRCs or nonLRCs (B). (C) Percentage of Pax7^+^ or MyoG^+^ LRCs and nonLRCs from postnatal (P10) muscle after p27KD (left), from adult muscle after p27KD (middle), and from adult muscle after p21KD (right). Data were averaged (*n*=3 mice per group) and expressed as mean±s.e.m. (*t*-tests, *P<0.05). (D) Muscle injury strategy. (E,F) Quantification of Pax7^+^ cells per single muscle fiber (E) and average muscle fiber size (F) from control or *p27* null uninjured (UI), primary injured (1°) or secondary injured (2°) muscle. (G,H) Quantification of Pax7^+^ cells per mm^2^ (G) and average muscle fiber size (H) from control or *p21* null uninjured (UI), primary injured (1°) or secondary injured (2°) muscle. (E-H) Data were averaged (*n*=3 mice per group) and expressed as mean±s.e.m. (*t*-tests, *P<0.05). (I) Control and *p27* null uninjured and 2° injured muscle stained for laminin, pan myosin heavy chain and with DAPI to quantify average muscle fiber size. Scale bar: 200 μm.

We next tested whether p27^kip1^ or p21^cip1^ is required for maintaining SCs in a more primitive state during proliferation. We analyzed FACS sorted SCs (>95% Pax7^+^; supplementary material Fig. S6C) from Ctrl, *p27* null and *p21* null adult mice *in vitro*. After 4 days in culture, *p27* null cultures had fewer Pax7^+^ cells and more MyoG^+^ cells than Ctrl, suggesting that p27^kip1^ is essential for maintaining the self-renewal potential of SCs (Fig. 7F). Consistent with this model, adult *p27* null muscle had fewer SCs than the Ctrl (Fig. 8E). In contrast to p27^kip1^, but consistent with a requirement for p21^cip1^ in muscle differentiation (Zhang et al., 1999b), *p21* null cultures had more Pax7^+^ cells and fewer MyoG^+^ cells than Ctrl (Fig. 7G); however, no change in satellite number was detected in adult *p21* null muscle compared with Ctrl (Fig. 8G). This suggests that p21^cip1^ promotes the lineage commitment of SCs without compromising their ability to occupy the niche.

Based on the hierarchical relationship observed between H2B-LRCs and H2B-nonLRCs and the loss of self-renewal potential of juvenile SCs in *p27* null mice, we predicted that p27^kip1^ function was restricted to LRCs. To test this, we used lentivirus constructs containing shRNA against *p27^kip1^* (p27KD) or scrambled shRNA (Ctrl) to target wild-type juvenile (P10) LRCs and nonLRCs from TetO-H2B-GFP mice (Fig. 8A; supplementary material Fig. S7A-C). After 4 days in culture, as anticipated, Ctrl-LRCs were less committed than Ctrl-nonLRCs (Fig 8C). In cultured p27KD-LRCs, Pax7 expression declined and MyoG increased to levels observed in Ctrl-nonLRCs (Fig. 8C). Consistent with our hypothesis, p27KD-nonLRC cultures were comparable to Ctrl-nonLRCs (Fig. 8C). These results demonstrate that p27^kip1^ is required specifically in early forming LRCs to maintain a primitive fate *in vitro*. Moreover, the primitive fate of adult LRCs was also dependent on p27^kip1^ levels (Fig 8B,C). We next examined the phenotype of adult LRCs and nonLRCs after p21^cip1^ knockdown (Fig. 8B,C; supplementary material Fig. S7D,E). In contrast to p27^kip1^ knockdown cultures, no effect on Pax7 or MyoG expression was observed in p21KD-LRCs. However, p21KD-nonLRCs maintained a higher percentage of Pax7^+^ cells than did Ctrl-nonLRCs, suggesting that p21^cip1^ normally functions to promote the commitment of nonLRCs. In summary, these data show that p27^kip1^ is required specifically in LRCs to maintain a primitive fate; by contrast, p21^cip1^ is required specifically in nonLRCs to promote lineage commitment.

To examine whether the absence of p21^cip1^ or p27^kip1^ would impact SC repopulation and differentiation *in vivo*, we performed repeated muscle injuries on Ctrl, *p21* null and *p27* null adult mice (Fig. 8D-I). We observed that, in *p27* null muscle, both SC number and fiber size declined progressively during successive bouts of repair. By contrast, SC number and fiber size were restored after injury in Ctrl muscle (Fig. 8E,F,I). In *p21* null mice, no effect of repeated muscle injury was observed on either SC number or fiber size (Fig. 8G,H; supplementary material Fig. S7F). Together, these data suggest that p27^kip1^ is essential to maintain the self-renewal potential of LRCs and for the retention of SC number and function in contexts of high tissue turnover.

## DISCUSSION

Label retention is a common feature of adult stem cells across different niches (Fuchs, 2009; Li and Clevers, 2010). In this regard, subsets of adult stem cells that spend more time in quiescence compared with their more proliferative counterparts possess long-term self-renewal potential (Wilson et al., 2008; Foudi et al., 2009; Chakkalakal et al., 2012).

H2B-GFP and BrdU have been used previously to study muscle stem cell proliferative kinetics (Schultz, 1996; Shinin et al., 2006; Chakkalakal et al., 2012; Rocheteau et al., 2012). In this study, both H2B-GFP and BrdU were used to study the proliferative output of SCs during postnatal maturation. Importantly, these approaches do not always give identical results. For example, we show that 6 weeks of Dox administration to adult TetO-H2B-GFP reporter mice labels nearly all quiescent SCs, whereas under the same period BrdU administration labels only 5-10% of SCs (see also Chakkalakal et al., 2012). In addition, H2B-GFP can be detected over 8 cell divisions, whereas BrdU detection is lost after 4 divisions (van der Wath et al., 2009). This might partially explain the discrepancy between H2B-GFP- and BrdU-based methods in defining the size of the label-retaining stem cell compartment (Shinin et al., 2006; Chakkalakal et al., 2012). The retention or loss of BrdU can also reflect asymmetric chromosome segregation within a single mitosis (Shinin et al., 2006; Conboy et al., 2007). Moreover, cells will incorporate BrdU during a single S phase, which would not lead to dilution of H2B-GFP. Most significantly, unlike BrdU, H2B-GFP allows for prospective isolation (based on fluorescence intensity) and subsequent functional analysis of cells of distinct proliferative histories.

During embryonic myogenesis, proliferative SC precursors are observed in the limb bud and begin to occupy the niche during fetal development (Relaix et al., 2004, 2005; Kassar-Duchossoy et al., 2005; Brohl et al., 2012). Lineage tracking confirms that embryonic Pax7^+^ cells give rise to both developing muscle fibers and the future adult SC pool (Lepper and Fan, 2010). Previous studies have demonstrated SC heterogeneity based on subsets of cells with distinct proliferative histories (Schultz, 1996; Shinin et al., 2006). Using a TetO-H2B-GFP reporter system to measure proliferative history, we observed that LRCs and nonLRCs could be identified at birth, were sustained during postnatal growth and re-established after injury. This argues that the LRC pool is actively set aside into a relatively dormant state in spite of the inductive environment necessary for the proliferation and fusion of Pax7^+^ cells in order to achieve muscle growth. Significantly, we find that LRCs and nonLRCs are born functionally equivalent, but transition to a hierarchical relationship during ontogeny. Therefore, subsets of stem cells and progenitors reside within the adult SC niche, all marked by Pax7, albeit functionally distinct according to their proliferative history.

Numerous reports demonstrate that transplanted SCs efficiently repopulate the niche and contribute to myofiber repair (Collins et al., 2005; Montarras et al., 2005; Cerletti et al., 2008; Boldrin et al., 2009; Sacco et al., 2010), with subsets organized in a hierarchical relationship and specialized to self-renew or differentiate (Kuang et al., 2007; Tanaka et al., 2009; Rocheteau et al., 2012). In the present study, we confirm that the transplanted SC pool is functionally heterogeneous and hierarchically organized. However, we cannot exclude the possibility that, under other contexts, nonLRCs can acquire some of the characteristics of LRCs. For example, early in postnatal life nonLRCs possess self-renewal potential that is lost abruptly during maturation. Whether this reflects the dynamic entry of LRCs into the nonLRC pool during rapid muscle growth or the interconversion of nonLRCs into self-renewing cells cannot be determined at present.

During disease pathogenesis in mdx mice, there is a preference for SCs to differentiate rather than self-renew (Heslop et al., 2000; Yablonka-Reuveni and Anderson, 2006). To date, the mechanisms explaining this phenomenon remain unknown. That LRCs from mdx muscle retain high functionality suggests that the diminution of the SC pool is not through loss of self-renewal capacity at the population level, but instead through a subset of SCs moving down the hierarchy, i.e. converting from LRCs to nonLRCs.

Adult SCs that are enriched for *Pax7* and either lack or express low levels of *Myf5* possess potent self-renewal potential compared with their more committed progeny that have increased *Myf5* and decreased levels of *Pax7* (Gayraud-Morel et al., 2007, 2012; Kuang et al., 2007; Rocheteau et al., 2012). In the present work, we observe that myogenic fate genes such as *Myf5* and *Pax7* are enriched only in adult LRCs, suggesting that Pax7^hi^ and LRC subsets might partially overlap. Genetic strategies have revealed that SCs from mice heterozygous for *Pax7* are functionally indistinguishable from wild-type SCs (Gayraud-Morel et al., 2012), arguing that phenotypic differences between adult LRCs and nonLRCs are unlikely to arise through a decrease in *Pax7* levels. In addition, although P21 LRCs and nonLRCs are functionally distinct, they do not however have different *Pax7* or *Myf5* levels. Therefore, the present result is consistent with lower *Pax7* levels in adult nonLRCs reflecting their more committed nature (Olguin and Olwin, 2004; Zammit et al., 2004; Tajbakhsh et al., 2009).

In both adult and aged muscle, LRCs are enriched for expression of the FGF inhibitor *Spry1* and the cell cycle inhibitor *p27^kip1^* (Chakkalakal et al., 2012)*.* We demonstrated previously that *Spry1* is required for a subset of adult SCs to self-renew in response to multiple rounds of injury, but is redundant for differentiation (Shea et al., 2010). Together, these data indicate that LRCs possess molecular brakes and differential sensitivities to growth factors that restrict entry into the cell cycle. In line with this, deletion of the cell cycle inhibitor *p27^kip1^* led to increased proliferation, as detected by loss of BrdU, and a reduction in the self-renewal potential of H2B-LRCs. That loss of p27^kip1^ did not impact the cell fate bias of H2B-nonLRCs argues for its importance for maintaining the self-renewal potential of primitive stem cells and redundancy in their more committed progeny. In HSCs, p27^kip1^ is required for maintenance of the adult stem cell pool, but dispensable for self-renewal potential, the latter possibly owing to compensation by p57^kip2^ (Matsumoto et al., 2011; Zou et al., 2011). It had previously been demonstrated that the p27 family members p21^cip1^ and p57^kip2^ could function cooperatively to control the cell cycle exit and differentiation of myogenic progenitors; however, their role in SC self-renewal remained unexplored (Halevy et al., 1995; Parker et al., 1995; Zhang et al., 1999b; Reynaud et al., 2000; Hawke et al., 2003; Messina et al., 2005). Importantly, we show that deletion of *p21^cip1^* did not lead to a change in the self-renewal potential of H2B-LRCs. Deletion of *p21^cip1^* did, however, maintain a more primitive character in H2B-nonLRCs, which was strikingly different from the role of p27^kip1^. The influence of cell cycle regulators on cell fate is perhaps not surprising considering the sensitivity of cells during G1 of the cell cycle, when cell fate decisions are made (Zhang et al., 1999a; Orford and Scadden, 2008; Lange et al., 2009). However, the finding that different cell cycle inhibitors regulate distinct subsets of SCs is unexpected and suggests a complex role of cell cycle inhibitors at discrete steps of the stem cell hierarchy. Rather than being mere sentinels of proliferation, it seems feasible that p21^cip1^ and p27^kip1^ play direct roles in cell fate control, as suggested in embryonic stem cells (Wang and Blelloch, 2009; Li et al., 2012) and neural stem cells (Marques-Torrejon et al., 2013; Porlan et al., 2013). At present, we do not know the downstream effectors of p27^kip1^ action within the SC hierarchy.

Overall, our data demonstrate that label-retaining SCs are essential for the formation and maintenance of a functional stem cell pool during conditions of tissue turnover. We provide evidence that a cell cycle inhibitor, p27^kip1^, plays a crucial role in preserving the relative dormancy and primitive lineage of label-retaining SCs.

## MATERIALS AND METHODS

### Animals

TetOP-H2B-GFP mice were backcrossed onto a C57BL6 background (Foudi et al., 2009). Mice null for *p27^kip1^* and *p21^cip1^* were obtained from Jackson Labs. Animals were housed and handled in accordance with the guidelines of the Massachusetts General Hospital and Sanford-Burnham Medical Research Institute Subcommittee for Animal Research.

### Fluorescence-activated cell sorting (FACS)

Mononucleated cells were isolated from muscle as described (Shea et al., 2010). Subsequently, cells were incubated with anti-Vcam1-PE (Invitrogen), anti-integrin α7-649 (AbLab), anti-mouse CD31-PE-Cy7 (BD Pharmingen), anti-mouse CD45-PE-Cy7 (BD Pharmingen) and anti-mouse Sca1-APC-Cy7 (BD Pharmingen). Myogenic cells were isolated by CD31^−^/CD45^−^/Sca1^−^/integrin α7^+^/Vcam1^+^. The gates selecting for integrin α7^+^/Vcam1^+^ (double positives, DPs) were set according to single color controls.

LRC and nonLRC SCs were collected by creating positive selection gates at the boundaries where the cell numbers reach a minimum across the total H2B-GFP intensity. The separation between the gates had to be varied depending on the distribution of the H2B-GFP intensity, while taking into account the Gaussian distribution of the LRC and nonLRC subsets, and placing the gates conservatively so that minimal or no overlap would occur. For detailed examples on how gates were placed (see the supplementary material). The fraction of LRCs and nonLRCs within the total SC population was determined using FlowJo (Tree Star).

To ensure reproducibility of H2B-GFP emission intensity between different samples and sorting times, the voltage of the photomultiplier receiving signal from the 488 nm laser was normalized using 6 μm PeakFlow Green Flow Cytometry Reference Beads (Life Sciences) immediately prior to every sort. Cells were sorted using a FACS Aria II (BD Biosciences).

### *In vivo* cell division analysis

Muscle SC number and fiber size were analyzed as described (Shea et al., 2010). To determine BrdU label retention, intraperitoneal injections of BrdU or EdU (6 μg/g body weight) were given twice daily from P3 to P7 followed by a 10-day chase. For P16 to P17 EdU *in vivo* pulsing, pups were administered three EdU pulses (6 μg/g body weight) over 24 h and sacrificed for FACS sorting 1.5 h later.

For transient H2B-GFP expression in adult mice, Dox (1 mg/ml; Sigma-Aldrich) was administered as previously described (Chakkalakal et al., 2012). For embryonic H2B-GFP expression, pregnant females were injected at E10.5 with Dox (150 μl at 1 mg/ml) and immediately switched to drinking water containing Dox (2 mg/ml) until E16, at which time drinking water was switched back to pure water. To activate H2B-GFP *in vitro*, Dox (100 μg/ml) was added to SC cultures for 18 h.

To obtain cells for manual H2B-GFP quantification in Pax7^+^ cells, mononucleated cells were incubated with biotinylated anti-mouse Sca1, anti-mouse CD31 and anti-mouse CD45 and negatively enriched for CD31^−^/CD45^−^/Sca1^−^ cells using streptavidin-conjugated magnetic beads according to manufacturer's instructions (Stem Cell Technologies). H2B-GFP intensities and background values were quantified by manually encircling Pax7^+^ nuclei and nearby background using Nikon Eclipse software. The H2B-GFP intensities were then subtracted for the background intensity, converted to logarithmic values and plotted with Microsoft Excel and Graph Pad Prism software.

### SC transplantation

LRCs and nonLRCs were sorted from skeletal muscles and transplanted as described previously (Chakkalakal et al., 2012). After the 30-day recovery, transplanted muscles were dissected, fixed in ice-cold 4% paraformaldehyde and incubated overnight in 30% sucrose solution. Transverse sections were obtained as described (Chakkalakal et al., 2012). The total numbers of sublaminar GFP^+^ nuclei and Pax7^+^ cells were counted for every 200 μm of muscle sectioned.

### Muscle injury and cell culture

Injury to whole TA/EDL muscle, single fiber isolation and analysis, muscle histology and immunofluorescence were performed as described previously (Shea et al., 2010). A longer incubation time (90 min) was required to isolate single fibers from mdx mice. Split fibers that occur as a consequence of disease progression were excluded from the analysis. SCs were fixed immediately or plated in plating medium (DMEM with 10% horse serum) on extracellular matrix (Sigma) for 3-4 days and stained for Pax7, Ki67, MyoD, MyoG, Myf5 and p27^kip1^ (described in full in supplementary material Table S1).

### RNA isolation and RT-PCR

RNA extraction from FACS sorted SCs was performed using Trizol (Invitrogen) with the manufacturer's suggested modification of the addition of ultrapure glycogen (Invitrogen) and prepared for quantitative real-time PCR (qRT-PCR) analysis on a StepOnePlus real-time PCR machine (Applied Biosystems) with Platinum SYBR Green qPCR SuperMix-UDG with ROX Master Mix (Invitrogen) using primers against *Pax7*, *Myf5*, *Myod*, *Spry1*, *p21^cip1^*, *p27^kip1^*, *p57^kip2^* and *Gapdh* (supplementary material Table S1). Unless otherwise stated, data were from separate reactions performed in quadruplicate from *n*=4-6 mice for each condition.

### Lentiviral infection

Sorted SCs (2500) were plated in plating medium, allowed to recover by incubation at 37°C in 5% CO_2_ for 16-18 h and subsequently infected at a MOI of 10 with lentivirus containing GFP, scrambled (Ctrl) or shRNA to *p21^cip1^* or *p27^kip1^* (Sigma-Aldrich) according to the manufacturer's protocol for 24 h. Cells were rinsed and incubated in fresh plating medium from 48 h to 4 days and fixed.

### Statistical analysis

A minimum of three and up to six replicates were performed for all experiments presented, unless otherwise stated. Data are presented as means with s.e.m. Comparisons between groups were performed using a one-way ANOVA and a Bonferroni post-hoc test. Comparisons within groups were undertaken using a *t*-test with repeated measures. Differences were considered statistically significant at *P*<0.05.

## Supplementary Material

Supplementary Material
