# Supplementary material for: Early forming label-retaining muscle stem cells require p27kip1 for maintenance of the primitive state
Source: Development. 2014 Apr;141(8):1649–59. doi: 10.1242/dev.100842 (PMC3978835; doi:10.1242/dev.100842)
Supplement: Supplementary Material [file supp_141_8_1649__index.html]

Supplementary Material 

# Early forming label-retaining muscle stem cells require p27kip1 for maintenance of the primitive state

## DEV100842 Supplementary Material

**Files in this Data Supplement:**

- **Supplementary Material**
