# Supplementary material for: Early forming label-retaining muscle stem cells require p27kip1 for maintenance of the primitive state
Source: Development. 2014 Apr;141(8):1649–59. doi: 10.1242/dev.100842 (PMC3978835; doi:10.1242/dev.100842)
Supplement: Supplementary Material [file supp_141.8.1649_DEV100842.pdf]

Determining positive selection gates for LRCs and nonLRCs

Positive selection gates for the collection of LRCs or nonLRCs had to be drawn based on the location and shape of the Gaussian distributions. For example, at the P1 time point (see Fig. 1B), distinct LRC/nonLRC peaks are less resolved and, therefore, to avoid cross-contamination the trajectory of the Gaussian nonLRC peak was extrapolated into the LRC peak, indicating where the population would have extended to if visible. The same was done for the LRC peak extending into the nonLRC peak. This way, the collection gate for nonLRCs was placed with a lower H2B-GFP intensity boundary of 200 ($10^{2,3}$) and an upper boundary of 2000 ($10^{3,3}$). The LRC collection gate was similarly placed with a lower H2B-GFP intensity boundary of 5000 ($10^{3,7}$) and an upper boundary of 30,000 ($10^{4,47}$). In adult regenerated muscle (see Fig. 2C), LRC and nonLRC peaks are well resolved and collection gates were placed as follows: nonLRCs were isolated by placing a gate with a lower GFP intensity boundary of 50 ($10^{1.7}$) and an upper boundary of 700 ($10^{2.85}$). LRCs were isolated by placing a gate with a lower intensity boundary of 1200 ($10^{3,08}$) and a upper boundary of 10,000 (10^{4}).

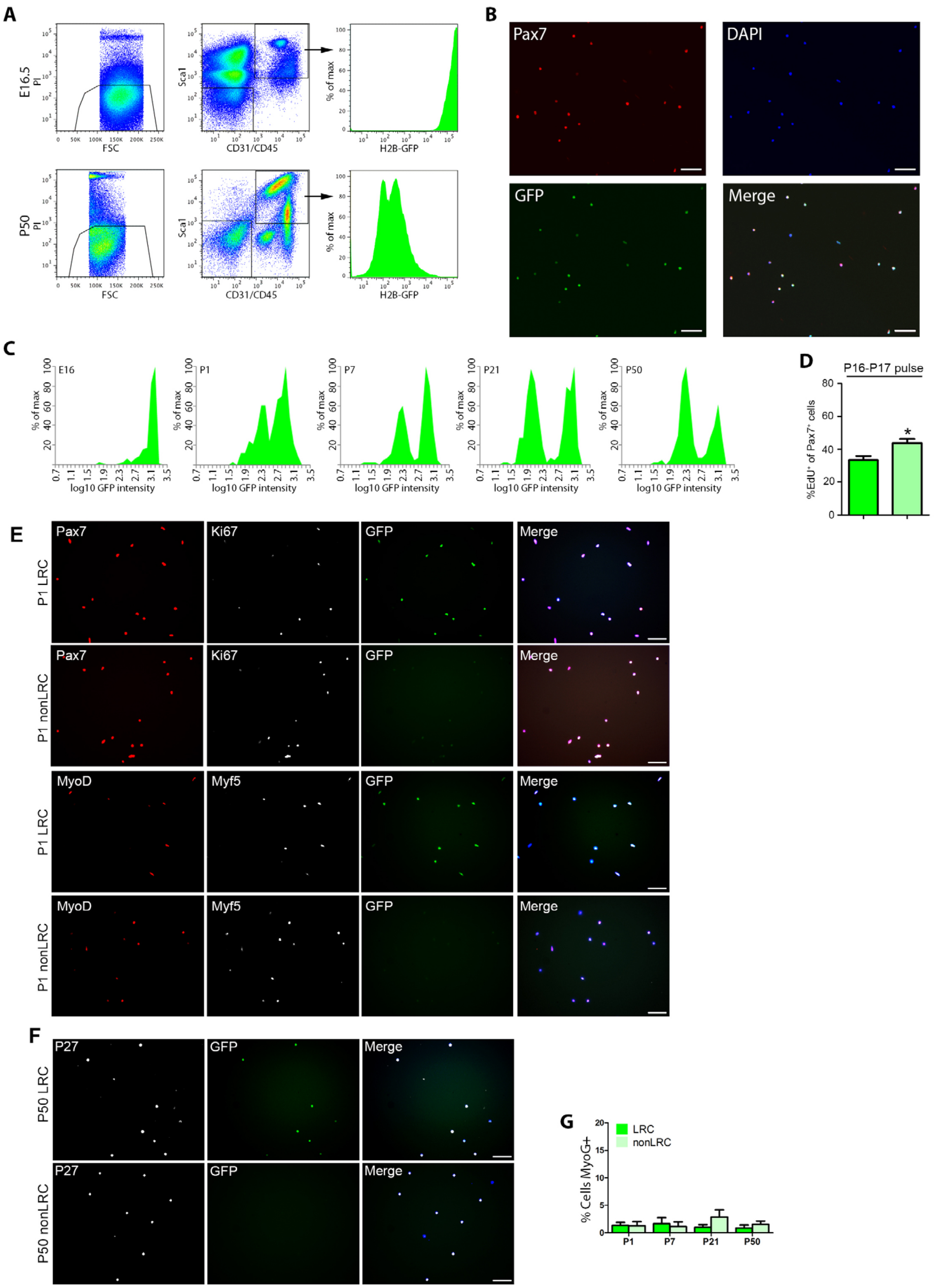

A
E16.5
PI
FSC
Sca1
CD31/CD45
% of max
H2B-GFP
P50
B
Pax7
DAPI
GFP
Merge
C
E16
P1
P7
P21
P50
% of max
log10 GFP intensity
D
P16-P17 pulse
%EdU+ of Pax7+ cells
*
E
P1 LRC
P1 nonLRC
Pax7
Ki67
GFP
Merge
MyoD
Myf5
F
P50 LRC
P50 nonLRC
P27
G
LRC
nonLRC
% Cells MyoG+
P1
P7
P21
P50

Supplemental Figure 1.H2B-GFP levels in satellite cells during ontogeny. (A) FACS sort profiles demonstrating H2B-GFP labeling (E16.5) and subsequent dilution of label (P50) in $CD31^+/CD45^+/Sca1^+$ cells. The $CD31^-/CD45^-/Sca1^-$ population (middle panel, lower left gate) was subsequently selected for Vcam1 and Integrin-α7 positivity (Fig. 1B). **(B)** Representative Pax7 staining (red) of freshly sorted H2B-GFP^+ (green) E16.5 myogenic cells. DAPI stains nuclei (blue). 90.0% ± 2.3% labeled positive for Pax7. **(C)** Mixed cell preparations obtained from muscle were enriched for satellite cells by negatively selecting for CD31, CD45 and Sca1 using antibody-conjugated magnetic bead, stained for Pax7 and quantified for H2B-GFP intensity (using Nikon Eclipse software). $Pax7^+$ cells separate into LRCs and nonLRCs from P1 onwards. **(D)** EdU retention in $Pax7^+$ sorted LRC or nonLRC satellite cells after P16 to P17 EdU pulse (3 pulses 12 hours apart). **(E)** Representative Pax7, Ki67, Myf5 and MyoD immunostaining of sorted LRCs and nonLRCs from P1 TetO-H2B-GFP mice. Scale bar represents 50 µm. **(F)** Representative p27 immunostaining of sorted LRCs and nonLRCs from P50 TetO-H2B-GFP mice. Scale bar represents 50 µm. **(G)** Percentage of sorted LRCs and nonLRCs staining positive for MyoG during ontogeny. Data were averaged (n=3 mice per group) and expressed as mean ± s.e.m.

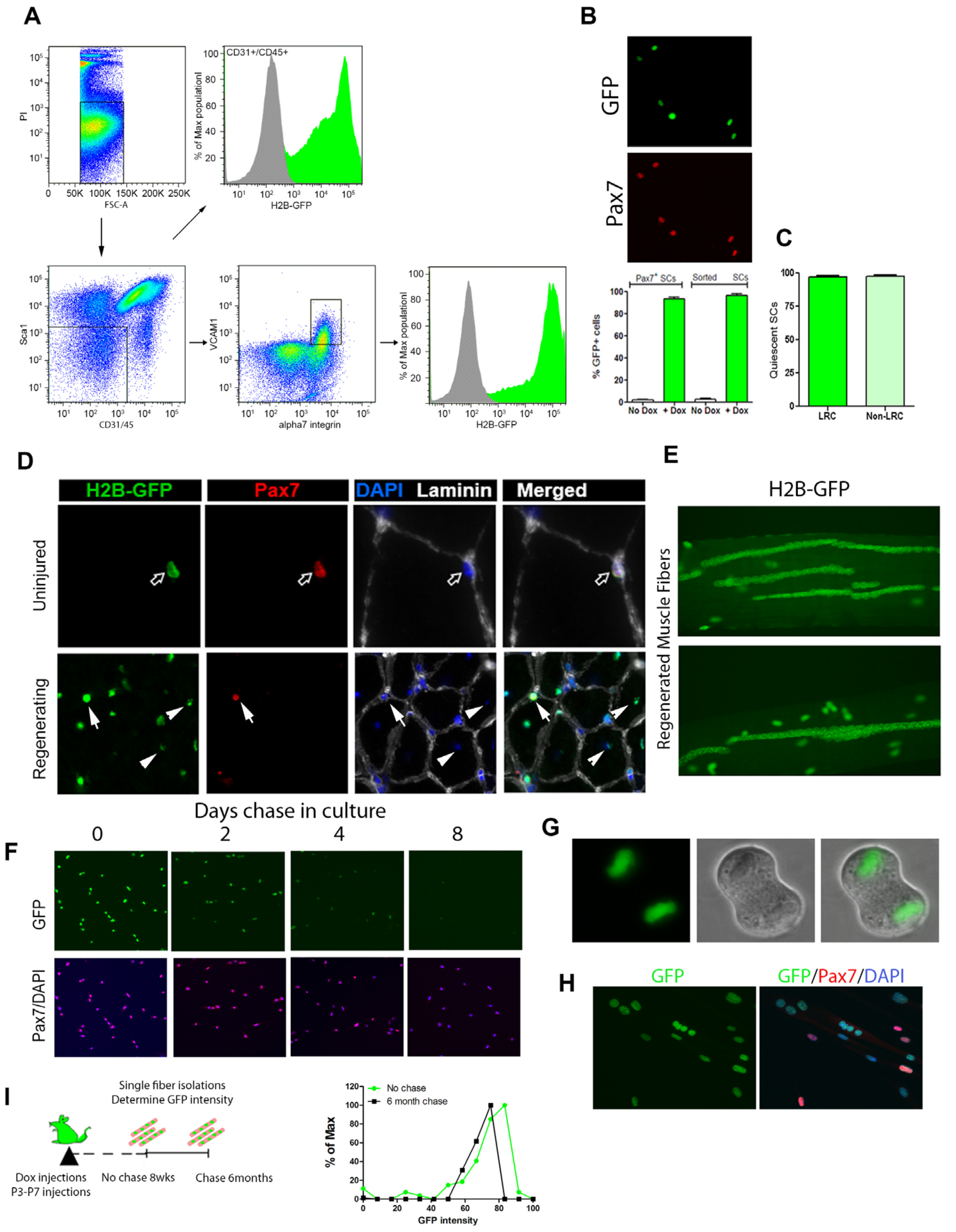
A
B
C
D
E
F
G
H
I
CD31+/CD45+
PI
FSC-A
% of Max populationl
H2B-GFP
Sca1
CD31/45
VCAM1
alpha7 integrin
GFP
Pax7
Pax7+ SCs
Sorted SCs
% GFP+ cells
No Dox
+ Dox
Quiescent SCs
LRC
Non-LRC
H2B-GFP
Pax7
DAPI
Laminin
Merged
Uninjured
Regenerating
Regenerated Muscle Fibers
Days chase in culture
0
2
4
8
Pax7/DAPI
GFP/Pax7/DAPI
Single fiber isolations
Determine GFP intensity
Dox injections
P3-P7 injections
No chase 8wks
Chase 6months
No chase
6 month chase
% of Max
GFP intensity

Supplemental Figure 2. H2B-GFP expression in satellite cell progenitors in vitro. (A) FACS sort profiles demonstrating H2B-GFP labeling in CD31$^+$/CD45$^+$ cells (upper panels) in adult mice fed Dox for 6 weeks in drinking water (no chase). The CD31$^-$/CD45$^-$ population (lower panels) was subsequently selected for Vcam1 and Integrin-α7 positivity from which GFP levels were assessed. **(B)** Image of Pax7 immuno-staining and H2B-GFP expression from freshly isolated satellite cells sorted by FACS and the percentage of satellite cells expressing GFP after Dox or vehicle treatment. Data were averaged (n=6 mice per group) and expressed as mean ± s.e.m. **(C)** Percentage of quiescent (Pax7$^+$/Ki67$^-$/MyoD$^-$) LRCs or nonLRCs isolated from 30 day regenerated muscle. Data were averaged (n=2 mice per group) and expressed as mean ± s.e.m. **(D)** H2B-GFP expression in uninjured and 30 day regenerated adult muscle after 6 weeks dox treatment. In uninjured muscle, arrows indicate H2B-GFP$^+$ labeling of a sublaminar satellite cell In regenerated muscle H2B-GFP can be detected in satellite cells (arrows) and central nuclei (arrowheads). The H2B-GFP signal is saturated compared to the less bright myonuclei. **(E)** Image shows H2B-GFP signal in centrally located 'nuclear chains' in single fibers from 30 day regenerated muscle. **(F)** H2B-GFP intensity decreases in Pax7 expressing satellite cells incubated in growth conditions for 8 days. **(G)** H2B-GFP is partitioned equally in sorted satellite cells undergoing cytokinesis. **(H)** Dox treated satellite cells incubated in low serum maintains H2B-GFP expression in Pax7$^+$ cells and myotubes after 4 days in culture. **(I)** Schematic of strategy to assess GFP dilution from terminally differentiated single muscle fiber nuclei and profile of GFP intensity from no chase and 6 month chased terminally differentiated muscle fiber nuclei.

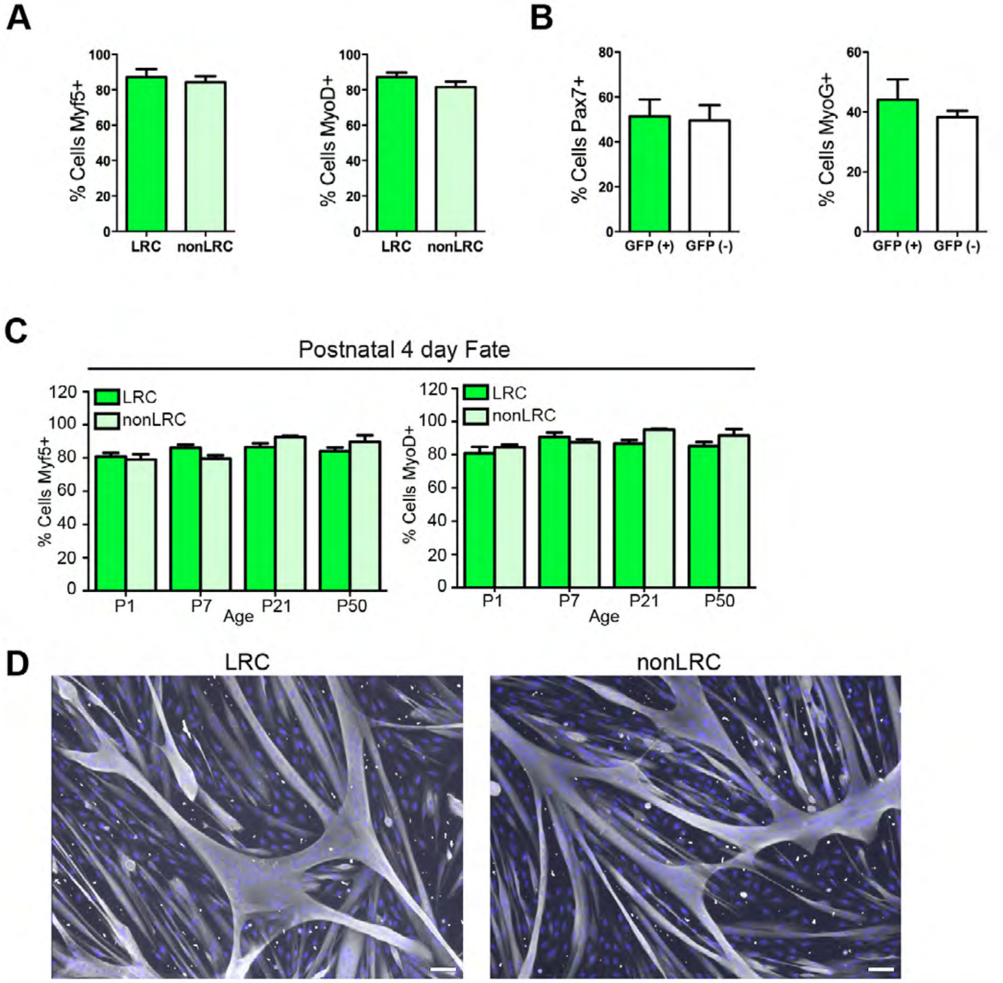

Supplemental Figure 3. LRCs can give rise to themselves and nonLRCs. (A) Percentage of sorted LRCs or nonLRCs from 30 day regenerated muscle cultured in plating medium for 4 days staining positive for Myf5 or MyoD. Data were averaged (n=4 mice per group) and expressed as mean ± s.e.m. **(B)** Percentage of sorted H2B-GFP labeled and rare unlabeled satellite cells after 6 weeks Dox treatment (no chase) cultured for 4 days and stained for Pax7 and MyoG. Data were averaged (n=2 mice per group) and expressed as mean ± s.e.m. Note, 4 day fate analysis indicates rare unlabeled cells are indistinguishable from H2B-GFP$^+$ satellite cells. **(C)** Percentage of sorted LRCs or nonLRCs isolated from indicated neonatal and postnatal stages and plated for 4 days staining positive for Myf5 or MyoD. Data were averaged (n=3 mice per group) and expressed as mean ± s.e.m. (t-tests, $P < 0.05$, LRCs vs nonLRCs). **(D)** Representative images of myotubes derived from 8500 LRCs and nonLRCs grown for 4 days (20% FBS, F10) and induced to differentiate for 2 days (3% HS, DMEM). Scale bars represents 50 μm.

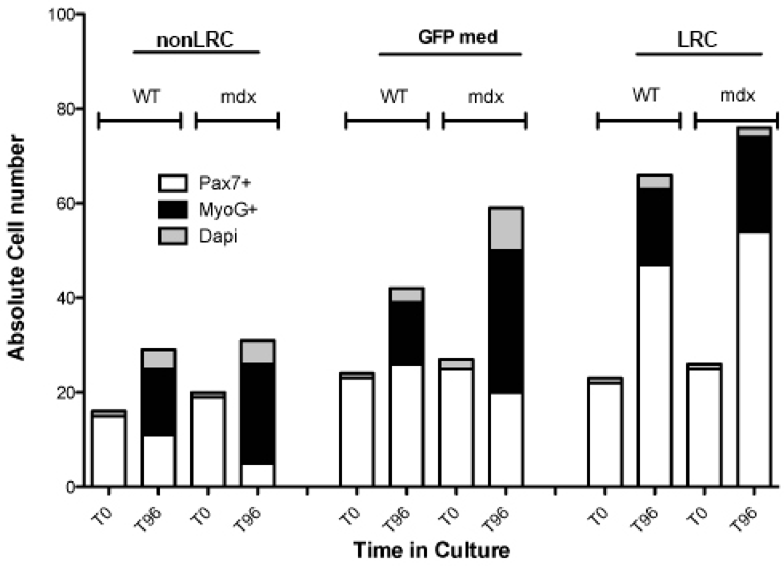

Supplemental Figure 4. Satellite cells from mdx mice are biased to differentiate in culture. Satellite cells were sorted according to H2B-GFP levels and fixed immediately (T0) or cultured in plating medium for 4 days (T96), fixed and stained for Pax7 (white bars) and MyoG (black bars).

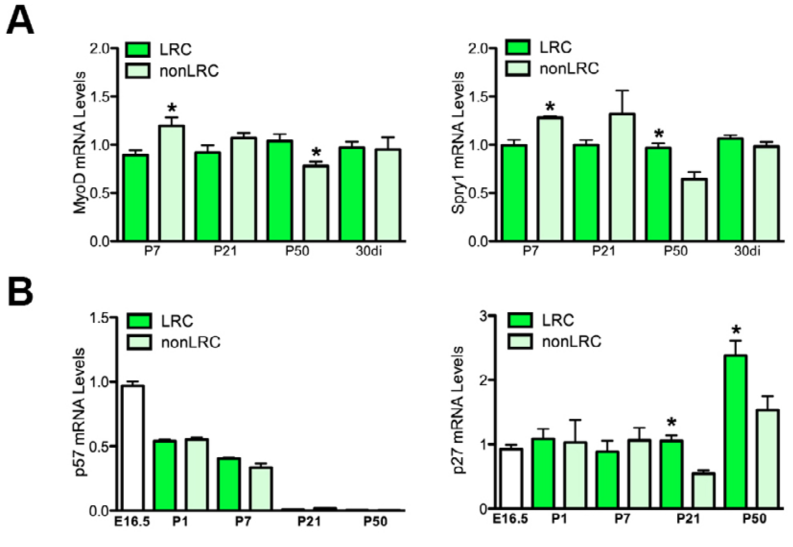

Supplemental Figure 5. Expression profile of cell fate markers and cell cycle inhibitors in LRCs and nonLRCs. (A) Expression of satellite cell fate markers (*myod* and *spry1*) in LRCs and nonLRCs during ontogeny and after injury. Expression was normalized to the corresponding gene expression level in LRCs at the P7 timepoint. The normalization within the P7 timepoint was obtained by setting the first of four replicates to 1. **(B)** Expression levels of $p57^{kip2}$ and $p27^{kip1}$ during ontogeny when normalized to expression levels at E16.5. The normalization within the E16.5 timepoint was obtained by setting the first of four replicates to 1. Note, the decline in $p57^{kip}$ levels at late stages of postnatal maturation. Data were averaged (n=3-4 mice per group) and expressed as mean ± s.e.m. ($P < 0.05$).

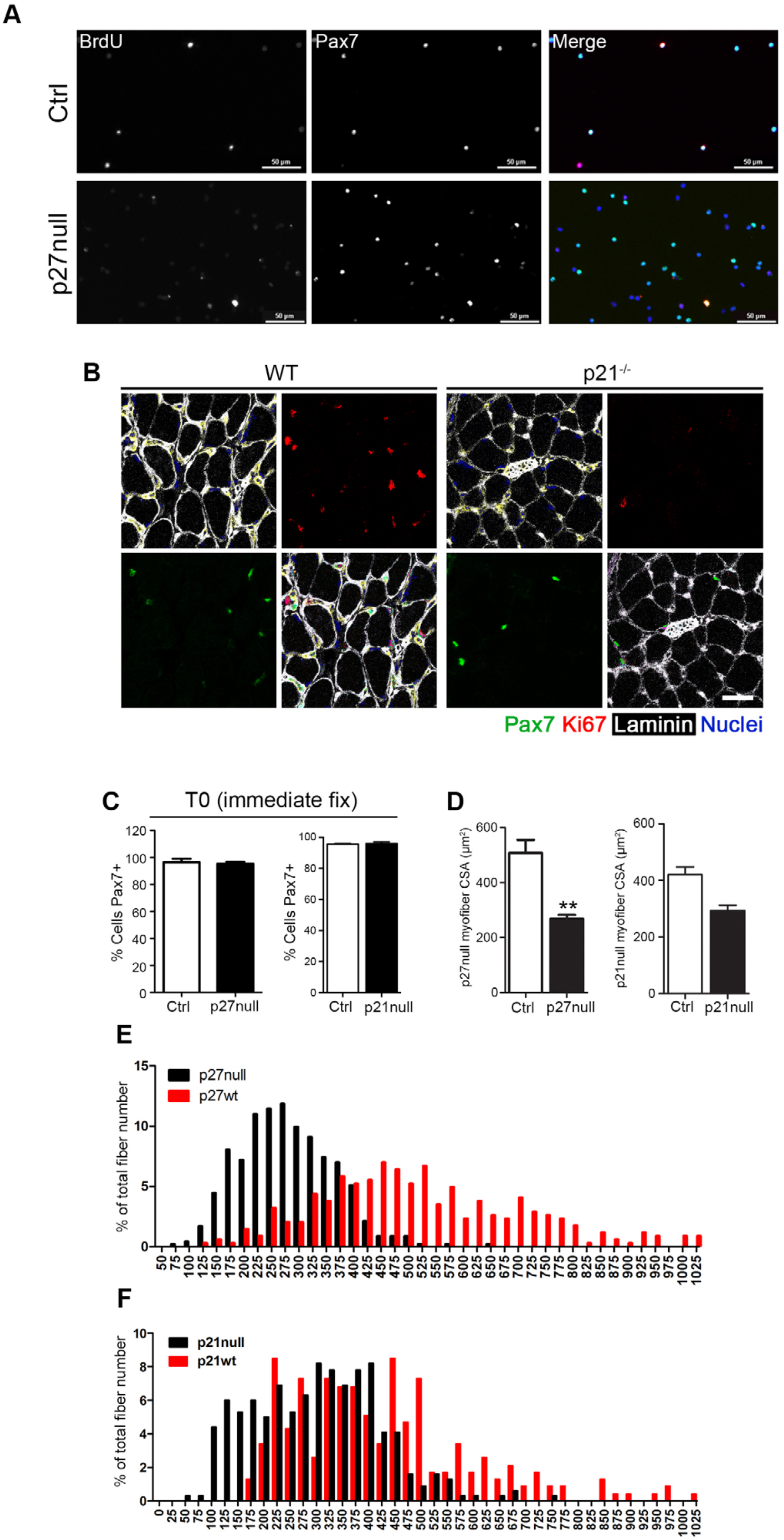
A
BrdU
Pax7
Merge
Ctrl
p27null
50 µm
B
WT
p21-/-
Pax7 Ki67 Laminin Nuclei
C
T0 (immediate fix)
% Cells Pax7+
Ctrl
p27null
p21null
D
p27null myofiber CSA (µm²)
p21null myofiber CSA (µm²)
**
Ctrl
p27null
p21null
E
p27null
p27wt
% of total fiber number
F
p21null
p21wt
% of total fiber number

Supplemental Figure 6. Muscle phenotypes in p21 and p27 germline knockouts.
(**A**) Representative images of sorted satellite cells from postnatal p27null muscles stained for BrdU+ and Pax7+. Scale bar represents 50 µm. **(B)** Representative images of Pax7+ and Ki67+ cells in postnatal p21null muscle sections. Scale bar represents 50 µm. **(C)** Sorted satellite cells from p21null, p27null or control littermates were plated down, fixed immediately, and immunostained for Pax7. >90% of sorted cells were Pax7+ in all conditions. **(D)** Quantification of myofiber cross sectional area (CSA) in EDL muscle from postnatal (P17) p27null, p21null or control littermates. Data were averaged (n=3 mice per group) and expressed as mean ± s.e.m. (** P < 0.01). **(E)** Representative histogram showing distribution of myofiber CSA in EDL muscle from postnatal (P17) p27null or control littermates. **(F)** Representative histogram showing distribution of myofiber CSA in EDL muscle from postnatal (P17) p21null or control littermates.

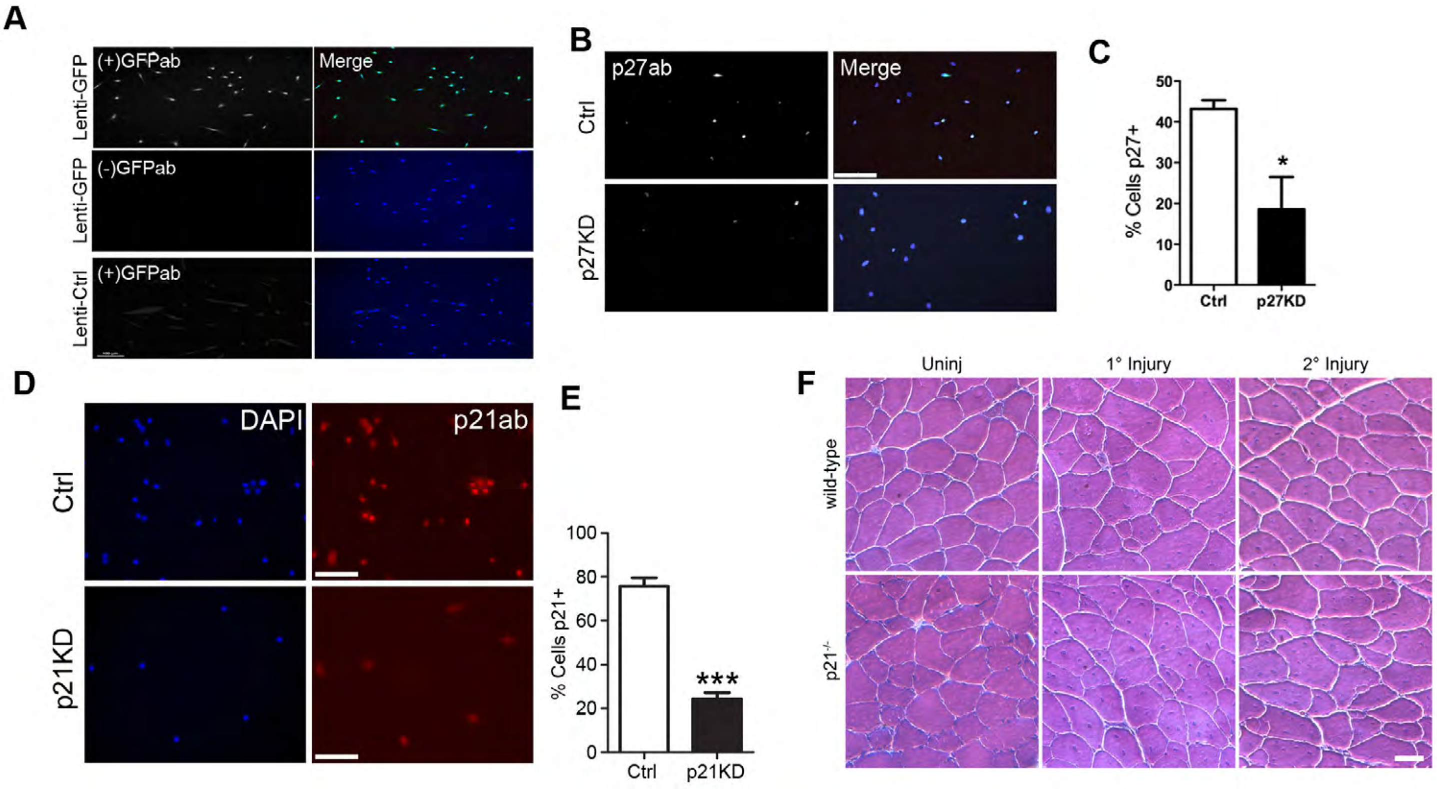

Supplemental Figure 7. shRNA mediated knockdown of P21 and P27 in LRCs and nonLRCs
(A) Representative images of lenti-viral mediated infection efficiency of satellite cells (10 MOI). Top two rows depict satellite cells infected with lenti-GFP and stained in the presence (+) or absence (-) of GFP antibody. Bottom row depicts satellite cells infected with lenti-ctrl (scrambled) and stained with GFP antibody. Lenti-viral infection is efficient and GFP staining is specific. **(B, C)** Representative images and efficiency of lenti-viral mediated siRNA knockdown of p27. Data were averaged (n=3-4 mice) and expressed as mean ± s.e.m. (* P < 0.05). Briefly satellite cells were sorted, plated for 16-18 hours prior to infection with lenti-p27 shRNA for 24 hours. Infected cells were cultured for an additional 48 hours prior to fixation and staining for p27 protein levels. **(D, E)** Representative images and efficiency of lenti-viral mediated siRNA knockdown of p21. Data were averaged (n=3 mice) and expressed as mean ± s.e.m. (*** P < 0.001). Briefly satellite cells were sorted, plated for 16-18 hours prior to infection with lenti-p21 shRNA for 24 hours. Infected cells were cultured for an additional 24 hours and switched to differentiation media for an additional 24 hours prior to fixation and staining for p21 protein levels. **(F)** Representative HE staining of control or p21null uninjured, primary injured or secondary injured TA+EDL muscle. Scale bar represents 50 µm.

Supplementary Table 1. List of antibodies and dilutions used as well as qPCR primers.

Antibody	Dilution	Vendor info
Mouse-anti-Pax7	1:100	Developmental Studies Hybridoma Bank
Mouse-anti-MyoD	1:150	BD Biosciences, Cat# 554130
Rabbit-anti-MyoD	1:100	Santa Cruz Biotechnology, Cat# sc-760
Rabbit-anti-MyoG	1:100	Santa Cruz Biotechnology, Cat# sc-576
Rabbit-anti-Myf5	1:100	Santa Cruz Biotechnology, Cat# sc-302
Rabbit-anti-Ki67	1:500	Abcam, Cat# ab66155
Rabbit-anti-p27	1:1	Abcam #ab15491
Mouse-anti-p27	1:100	BD biosciences, Cat#610241
Rabbit-anti-p21	1:50	Abcam, Cat#Ab2961
Rat-anti-BrdU	1:500	Abcam, Cat#Ab6326
Chicken-anti-laminin	1:3000	Abcam, Cat#Ab14055
Rabbit-anti-Cleaved Caspase-3	1:500	Cell Signaling Technologies, Cat#96615
Rabbit-anti-GFP	1:500	Invitrogen, Cat#A11122
Goat-anti-mouse 488	1:2000	Invitrogen #A11029
Goat anti-rabbit 488	1:2000	Invitrogen #A11008
Goat anti-mouse 546	1:2000	Invitrogen #A11003
Goat anti-rabbit 546	1:2000	Invitrogen #A11035
Goat anti-mouse 647	1:2000	Invitrogen #A21236
Goat anti-rabbit 647	1:2000	Invitrogen #A21245
Goat anti-rat 647	1:2000	Invitrogen #A21247
PE-conjungated Ms-anti-VCAM1	1:100	Invitrogen, Cat#RMC10604
647-conjugated Ms-anti-Integrin α7	1:100	Ablab, Cat#AB0000442
PE-CY7-conjugated Ms-anti-CD31	1:200	BD Pharmingen, Cat#561410
PE-CY7-conjugated Ms-anti-CD45	1:200	BD Pharmingen, Cat#552848
APC-CY7-conjugated Ms-anti-Sca1	1:200	BD Pharmingen, Cat#560654
DAPI	1:2000	Invitrogen, Cat#D3571

Primer target	Forward primer (5’-3’)	Reverse primer (5’-3’)
Pax7	GTGGAATCAGAACCCGACCTC	GTAGTGGGTCCTCTCAAAGGC
Myf5	ATTACAGCCTGGCGGGACAGAG3	GCAATCCAAGCTGGACACGGAG
MyoD	CACTACAGTGGGGACTCAGATGC3	TCGACACAGCCGCACTCTTC
Spry1	GAGGCCGAGGATTTCAGATGCA	CTGAATCACCACTAGCGAAGTGT
p21	TGGAGTCAGGCGCAGATCCAC	CGCCATGAGCGCATCGCAATC
p27	AGGCAAACTCTGAGGACCGGCA	TGCTCCACAGTGCCAGCGTTC
p57	CGAGGAGCAGGACGAGAATC	GAAGAAGTCGTTCGCATTGGC
Gapdh	AGGTCGGTGTGAACGGATTTG	TGTAGACCATGTAGTTGAGGTCA
